# H_2_S Persulfidated and Increased Kinase Activity of MPK4 to Response Cold Stress in Arabidopsis

**DOI:** 10.3389/fmolb.2021.635470

**Published:** 2021-03-11

**Authors:** Xinzhe Du, Zhuping Jin, Zhiqiang Liu, Danmei Liu, Liping Zhang, Xiaoli Ma, Guangdong Yang, Sha Liu, Yarong Guo, Yanxi Pei

**Affiliations:** ^1^ Department of Psychiatry, Firtst Hospital/First Clinical Medical College of Shanxi Medical University, Taiyuan, China; ^2^ School of Life Science, Shanxi University, Taiyuan, China; ^3^ Department of Chemistry and Biochemistry, Laurentian University, Sudbury, ON, Canada

**Keywords:** hydrogen sulfide, Persulfidation, MPK4, MEK2, cold stress

## Abstract

Hydrogen sulfide (H_2_S) is a gasotransmitter along with nitric oxide and carbon oxide, which is involved in plant growth and development as well as biotic and abiotic stress resistance. In a previous study, we reported that mitogen-activated protein kinases, especially MPK4, are important downstream components of H_2_S involved in alleviating cold stress; however the underlying mechanism is unclear. In this study, we determined that the ability of H_2_S to alleviate cold stress is impaired in *mpk4* mutants, but not in the upstream *mek2* and *crlk1* mutants. MPK4 was basically persulfidated, and NaHS (H_2_S donor) further increased the persulfidation level of MPK4. MEK2 was not persulfidated by H_2_S. NaHS treatments increased the MPK4 activity level nearly tenfold. The persulfidation signal of MPK4 did not disappear after eight cystein residues in MPK4 were site-mutated, respectively. Above all, our results suggested that H_2_S alleviates cold stress directly by persulfidating MPK4 and increasing the MPK4 kinase activity.

## Introduction

Hydrogen sulfide (H_2_S) is a colorless and combustible gas with an odor similar to that of rotten eggs. H_2_S is toxic at high concentrations; however, at low concentrations, H_2_S plays important roles in both animals and plants as a gasotransmitter, along with nitric oxide and carbon oxide (Wang, 2012; Wang, 2014). H_2_S participates in the plant growth and development, as well as biotic and abiotic stress responses, including those to cold and drought. In proteins, H_2_S modifies cysteines (Cys) by persulfidation, which alters the -SH of Cys to -SSH, to regulate the activity or cell location of a protein. Persulfidation was first reported in mice, in which about 10%–25% of liver proteins, including glyceraldehyde-3-phosphate dehydrogenase (GAPDH), actin and tubulin, are persulfidated under physiological conditions. Persulfidation increases the activtiy of GAPDH and actin polymerization (Mustafa et al., 2009). In plants, there are also reports of persulfidated proteins (Aroca et al., 2015; Aroca et al., 2017). Aroca et al. reported that 2015 proteins in Arabidopsis seedlings were persulfidation. They accounted for 5% of the total proteins that participate in important plant processes, such as carbon metabolism, responses to abiotic and biotic stresses, growth and development, and RNA translation (Aroca et al., 2017).

Mitogen-activated protein kinases (MAPKs) are crucial components of cell signaling transduction in eukaryotes. MAPKs consist of three cascades: MEKK, MEK and MPK which phosphorylate and thereby activated next cascade respectively. When the cell membrane received the signal from outside, the receptor on the membrane was activated firstly, then MEKK→MEK→MPK cascades was activated, MPK activated downstream target such as enzymes, transcription factor leading to cell response (Šamajová et al., 2013). MAPK is involved in a wide range of physiological processes in plants including growth and development, and biotic and abiotic stress responses. MEKK1-MEK2-MPK4 plays a positive role in cold- and salt-stress responses in Arabidopsis (Teige et al., 2004). CRLK1, a calcium/calmodulin-regulated receptor-like kinase located in the plasma membrane, is the upstream of this cascade and interacts with MEKK1. Consequently, MEKK1 is phosphorylated when CRLK1 is activated by calcium signals under cold-stress conditions (Yang et al., 2010a; Yang et al., 2010b; Furuya et al., 2013).

Cold stress is one of the common environmental stresses for plants, and has many adverse effects on plant, which affects the physiological function of plant cell, restricts plant growth and development, and even causes plant death. It is meaningful to study how plants respond to cold stress (Ding et al., 2019). H_2_S plays important role in plant response to cold stress. In a previous study we found that MAPKs especially MPK4 are important downstream components in alleviating cold stress. MPK4 is required for H_2_S to alleviate damage to roots caused by cold stresses and regulate stomatal movement. However, the mechanisms underlying these functions remain unknown (Du et al., 2019). Here, we first studied the ability of H_2_S alleviate cold stress in *mpk4*, *mek2* and *crlk1* mutants using root-tip bending experiments. The effects of H_2_S on the persulfidation of MEK2 and MPK4 are assessed by biotin-switch assays revealed that MPK4, not MEK2, is persulfidated by H_2_S. Consequently, we determined the effects of H_2_S on the kinase activity of MPK4. Finally we investigated the persulfidation signal of Cys-site mutanted MPK4 to determine which Cys residues are important for persulfidation.

## Materials and Methods

### Plant Materials and Growth Conditions

A Ds insertion mutant of *mpk4* of Landsberg background (Ler) was kindly provided by John Mundy of Copenhagen University, in which Ds was integrated eight nucleotides upstream of the first intron of MPK4, and the expression of MPK4 was not detectable (Petersen et al., 2000). A T-DNA insertion mutants of *mek2* (Garlic-511-H01.b.1a.Lb3Fa) of Columbia background (Col) (Teige et al., 2004) was provided by Markus Teige of Vienna University. A T-DNA insertion mutants of *crlk1* (SALK-016240C) of Col (Yang et al., 2010a) was bought from ABRC. Wild type (WT) Arabidopsis of Ler and Col were used as control, respectively. The primers for genotyping are listed in Supplementary Table S1.

The growth conditions were described previously (Jin et al., 2011). Briefly, the seeds were grown in the pots containing a 1:1:1 soil:perlite:vermiculite (v:v:v) mixture or on the plates containing 1/2 Murashige and Skoog (MS) medium after been sterilizing with 75% ethyl alcohol and 6% NaClO. The seedlings were grown at 23°C, with 60% relative humidity and a 16/8 h (light/dark) photoperiod with 160 μEmm^−2^s^−1^ light illumination.

### Root Tip-Bending Experiment

The root tip-bending experiments were performed as previously described (Du et al., 2017). Briefly, the seedlings were grown on 1/2 MS plates for 7 days. The treatment group was fumigated with 5 μM NaHS (H_2_S donor), while the control group was not fumigated. Then, the seedlings were transferred to new 1/2 MS plates and put into cold (4°C) or normal (23°C) conditions vertically and inversely. All the growth condition parameters of growth except temperature were the same.

### Molecular Cloning and Construction of Expression Vectors

The CDS of *MEK2* was PCR amplified and cloned downstream of a GST tag using EcoRI and XhoI restriction site in pGEX4T1. The CDS of *MPK4* was PCR amplified and cloned downstream of a His6 tag using NheI and BamHI restriction sites in pET28a. For site-mutated MPK4 and MEK2EE, primers with corresponding mutated sites (PM) were designed, PM/PF (forward primer of CDS) and PM/PR (reverse primer of CDS) were used to generate the mutant PCR fragment using the pET28a-MPK4 or pGEX4T1-MEK2 plasmid as the template. It was important to determine whether to use PM or its complement. Then, two PCR products were used as templates to generate the mutated MPK4 or MEK2EE using the PF/PR as primers. Cystein was substituted with alanine in MPK4, and MEK2EE were generated by changing both putative phosphorylation sites to glutamate residues (T220E and T226E), which resulted in a constitutively active kinase. The vectors were transformed into *Escherichia coli* DH5α competent cells, and the sequences were confirmed by sequencing. The primers used are listed in Supplementary Table S1

### Recombinant Protein Expression and Detection

pGEX4T1-MEK2 and pGEX4T1-MEK2EE were transformed into *E. coli* strain JM109. pET28a-MPK4 and its Cys-mutant vector were transformed into *E. coli* strain BL21. A single clone was incubated in 5 ml LB liquid medium supplemented with the appropriate antibiotics overnight at 37°C. Then, 2 ml solution was transferred into 100 ml LB liquid medium supplemented with appropriate antibiotics and incubated approxiamately 2.5 h at 37°C until the OD600 was 0.6–0.8. Then, 0.1 mM isopropyl β-D-1-thiogalactropyranoside was added to induce protein expression, and the *E. coli* cultures harboring MEK2 and MPK4 were incubated approximately 20 h at 28°C and 16°C, respectively. MEK2 and MEK2EE were purified using GST Selfinose resin and the MPK4 and Cys-mutant MPK4 were purified using Ni-NTA Sefinose resin. The purified protein was mixed with loading buffer containing *β*-mercaptoethanol and boiled for 10 min. 10% SDS-PAGE gel electrophoresis was performed and stained the gel with Coomassie Brilliant Blue.

### Biotin-Switch Assay

The methods were described previously (Mustafa et al., 2009). Briefly, 0.2 mg purified protein was treated with 0-2000 μM NaHS as indicated in the legend at 4°C for 30 min. Then, acetone was added, and samples were incubated −20°C for 20 min to precipitate the proteins. The proteins were resuspended in 100 μL HEN buffer (250 mM Hepes-NaOH, pH 7.7, 1 mM EDTA and 0.1 mM neocuproine) after acetone was removed. Then 400 μL 20 mM methyl methanethiosulfonate (MMTS) buffer, which had been dissolved with HEN buffer with 2.5% SDS, were added, and samples were incubated at 50°C for 25 min with frequent vortexing to block free -SH. Acetone was added, and the samples were placed at −20°C for 20 min to remove the MMTS and precipitate the proteins. The 100 μL HEN buffer with 1% SDS was used to resuspend the proteins, followed by the addition of 30 μL 2 mM biotin-HDPD in dimethyl sulfoxide to label the -SSH of Cys. After incubation for 3 h at 25°C, acetone was added to remove the biotin-HDPD and precipitated the proteins. Proteins were resuspended in HEN buffer and loading buffer without β-mercaptoethanol was added. After boiling for 10min, the samples were subjected to 10% SDS-PAGE gel electrophoresis, in which Selstain SDS-PAGE gel was used. Protein bands could be observed under UV irradiation in Selstain SDS-PAGE gel and served as an indication of loading. Then the proteins were transferred to nitrocellulose membrane, and subjected to Western blotting. A biotin antibody was used in Western blotting. The persulfidation to load ratio was then densitometrically analyzed with the software ImageJ.

### Kinase Activity Assay

His-MPK4 (0.2 μg) was activated by incubating with MEK2EE (0.05 μg) in a reaction buffer (20 mM Tris-HCl, pH 7.5, 10 mM MgCl_2_, and 1 mM DTT) containing 50 μM ATP at room temperature for 0.5 h. Activated MPK4 was treated with NaHS at 4°C for 30 min and then incubated with MBP (1:10 enzyme substrate ration) in the reaction buffer containing 50 μM ATP at room temperature for 0.5 h. The control was not treated with NaHS. The MPK4 activity was calculated based on the conversion of ATP to ADP using an ADP-Glo Kinase Assay (Promega).

### Statistical Analysis

Data are presented as the means ± standard error (SE). The determination of significant differences was performed with SPSS version 17.0 (SPSS, IBM, Chicago, IL, United States) using one way analysis of variance (ANOVA) followed by Duncan’s test or student’*t*-test as indicated in the figure legends. Different letters and asterisks represented significant differences among treatments. At least three independent experiments were performed.

## Results

### H_2_S Alleviates Cold Stress in an MEK2-Independent and MPK4-Dependent Way

**FIGURE 1:**
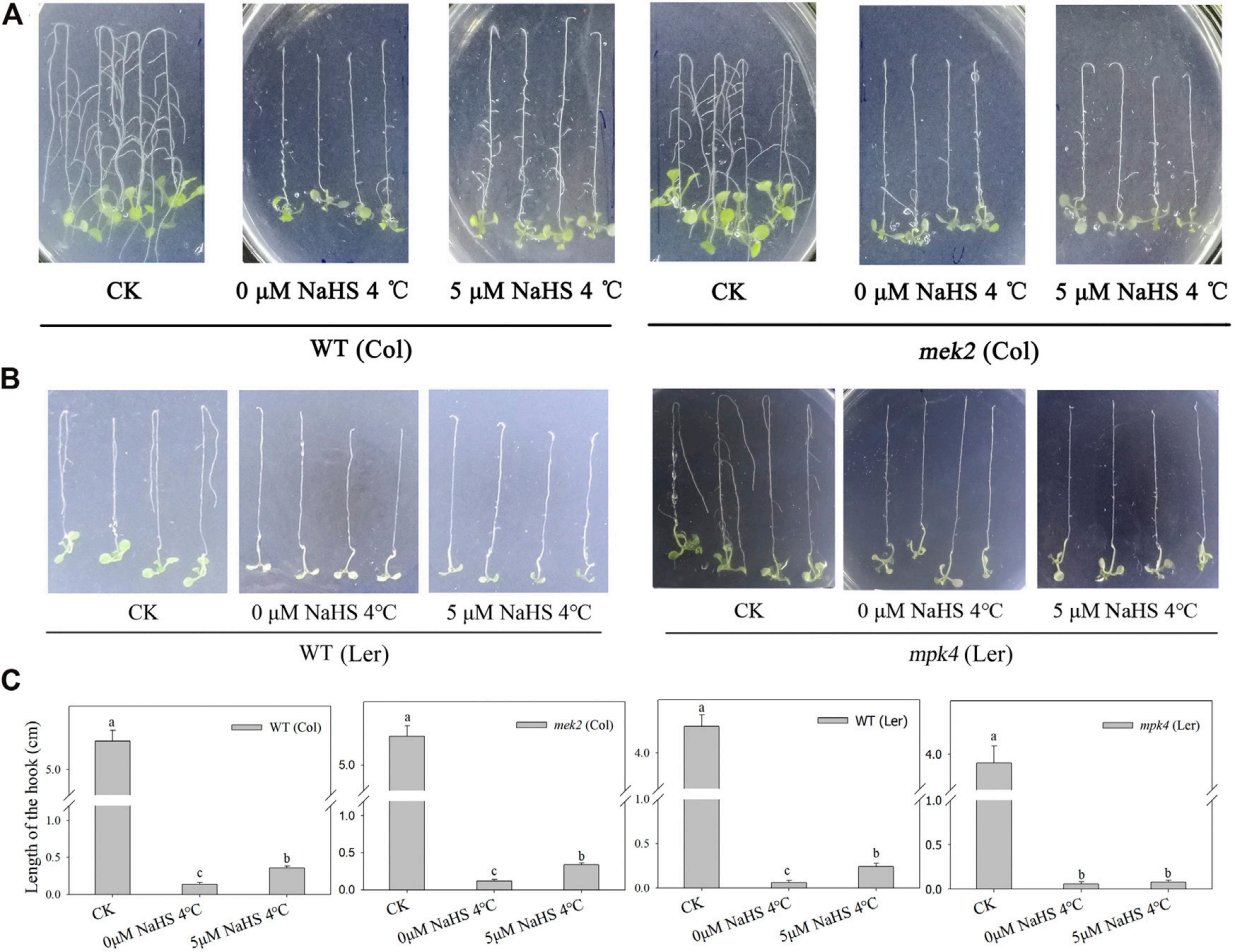
The alleviation effect of H_2_S in WT and *mek2* **(A)**, and *mpk4* **(B)** under cold stress. **(C)**. the quantitative analysis of hook of root bending in a and b. 1-week-old seedlings that grown on the 1/2 MS plates were fumigated by 5 μM NaHS for 12 h or not before transferred to 4°C inverserly. CK was still grown in 23°C inversely. The hook of root tip bending was observed 4 days later. Data are Mean ± SE and three independent experiment were repeated, different letters in c indicated significant differences among treatments (one way ANOVA *p* < 0.05).

In a previous study, we found that the function of H_2_S’s alleviation of cold stress requires MPK4. To determine whether H_2_S functions directly through MPK4 or MEK2, the cascade upstream of MPK4, we performed root tip-bending experiments using homozygous *mek2* (Col) and *mpk4* (Ler) seedlings (Supplementary Figure S1), and WT (Col) and WT (Ler) were used as control, respectively. Cold stress inhibits the growth of roots of WT, *mek2* and *mpk4* seedlings. NaHS pre-treatments alleviated the impairment of root growth caused by cold stress in WT and *mek2* seedlings, but not in *mpk4* seedlings (Figure 1).

CRLK1-MEKK1-MEK2-MPK4 is a known cascade in cold-signal transduction, and we obtained homozygous *crlk1* (Col) seeds (Supplementary Figure S1) and performed root tip-bending experiements to confirm the targets of H_2_S—mediated cold stress alleviation. NaHS alleviated the damage caused by 4°C treatments in both WT and crlk1 (Col) seedlings (Supplementary Figure S2).

### MPK4, but not MEK2, is Persulfidated

**FIGURE 2:**
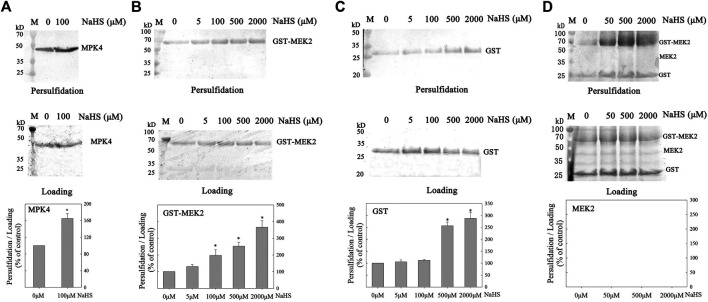
The effect of H_2_S on persulfidation of MPK4 and MEK2. The effect of H_2_S on persulfidation of MPK4 **(A)**, GST-MEK2 **(B)**, GST **(C)** and MEK2 **(D)**. MPK4, GST-MEK2, GST and MEK2 were incubated with NaHS of different concentration as indicated in the Figure in 4°C for 30 min, and subjected to biotin-switch assay, in which 10% SDS-PAGE gel and biotin antibody were used. Quantification anlysis was performed using ImageJ. Data are Mean ± SE, and three independent experiment were repeated. *indicated significant differences compared with control (Student’ *t*-test, *p* < 0.05). M: Marker.

H_2_S modifies the proteins through persulfidation to regulate the proteins’ function. Consequently, we investigated the persulfidation of MPK4 and MEK2. Firstly, we cloned the CDS sequence of *MPK4* into the pET28a vector, purified recombinant His-tagged MPK4 (41.36 kD, Supplementary Figure S3) and performed biotin-switch assays. MPK4 was basically persulfidated and NaHS treatments further increased MPK4 persulfidation (Figure 2A).

Then, we constructed the GST-tagged MEK2 cDNA plasmid pGEX4T1-MEK2 (Supplementary Figure S4A) and transfected it into *E. coil* from which recombinant GST-MEK2 was purified (65.93 kD, Supplementary Figure S4B). The GST-MEK2 was subjected to a biotin-switch assay. The NaHS treatment significantly increased the persulfidation level of GST-MEK2, and NaHS induced GST-MEK2 persulfidation in a concentration-dependent manner (Figure 2B). Because GST contains Cys residues, we then detected the persulfidation level of GST (26 kD) and found that it was also persulfidated by NaHS (Figure 2C). To exclude interference by the GST-tag, we digested the GST-MEK2 with Thrombin, which separates GST from MEK2, leaving the MEK2 protein (39.93 kD, Supplementary Figure S4B). Then, the persulfidation level of MEK2 was analyzed. MEK2 itselft didn’t show persulfidation signals and the NaHS treatment did not increase the persulfidation of MEK2 (Figure 2D).

### H_2_S Induces MPK4 Activity

**FIGURE 3:**
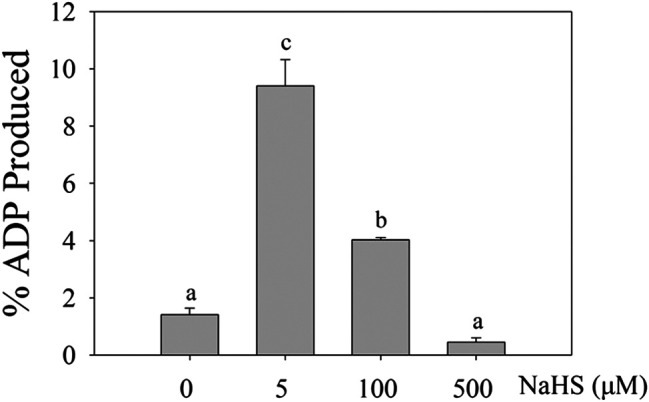
The effect of H_2_S on the activity of MPK4. The 0.2 μg purified MPK4 was first activated by 0.05 μg purified MEK2EE, then treated with 0 μM, 5 μM, 100μM and 500 μM NaHS in 4°C for 30 min. Activated MPK4 was incubated with MBP (1:10 enzyme substrate ration), and the activity of MPK4 was calculated by the conversion of ATP to ADP using ADP-Glo Kinase Assay (Promega). Data are Mean ± SE and three independent experiments were repeated, different letters indicated significant differences among treatments (one way ANOVA *p* < 0.05).

Then, we determined the role of H_2_S in the MPK4 activity using ADP-Glo agent, which allowed the kinase activity to be indicated by the ATP-ADP conversion ratio. The ADP produced in the kinase reaction increased after 5 μM and 100 μM NaHS treatments, indicating that the MPK4 kinase activity level increased after the NaHS treatment (Figure 3).

### The Persulfidation of MPK4 did not Disappeared After Cys Residues was Mutated

**FIGURE 4:**
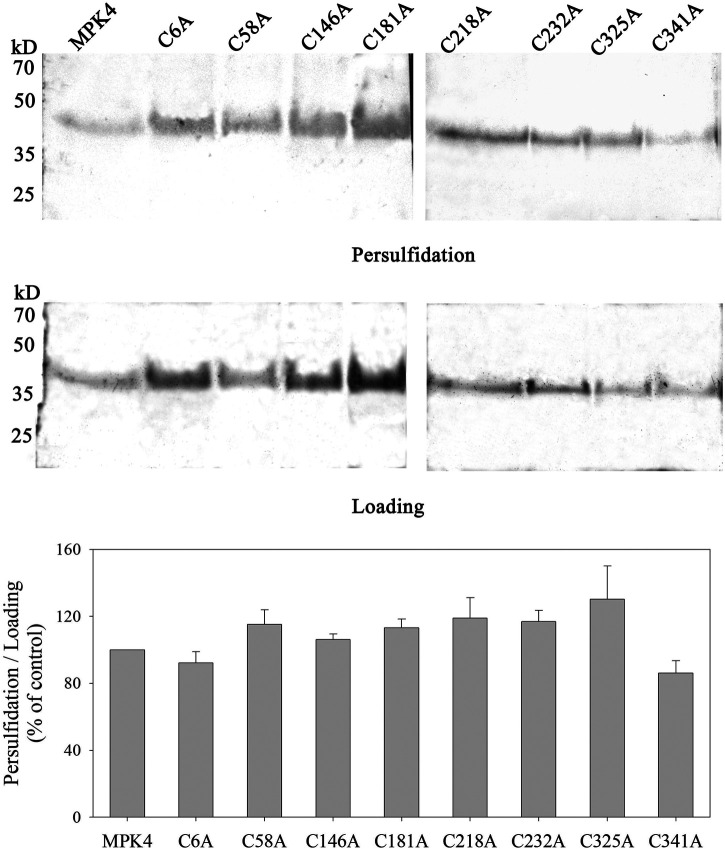
Persulfidation signal of MPK4 with and without Cys mutation. The eight Cys residues in MPK4 were site-mutated to alanine including cysteine 6, 58, 146, 181, 218, 232, 325 and 341, WT MPK4 protein and site-mutated MPK4 proteins were subjected to biotin-switch assay, in which 10% SDS-PAGE gel and biotin antibody were used. Quantification anlysis was performed using ImageJ. Data are Mean ± SE, and three independent experiment were repeated. The significance of difference was analyzed by student’ *t*-test. M: Marker.

Persulfidation modifications occur on the Cys residues, therefore, we mutated Cys residues in MPK4 to determine the important Cys residues involved in the persulfidation of MPK4. There are eight Cys residues in MPK4 (at the 6^th^, 58^th^, 146^th^, 181^st^, 218^th^, 232^nd^, 325^th^, and 341^st^ positions), and all of them were site-mutated to alanine, respectively. Then, the biotin-switch assay was performed. However, the persulfidation signal was detected in all eight Cys residues in MPK4, which had no significant difference with WT MPK4 (Figure 4).

## Discussion

As a gas signal molecule, H_2_S has many physiological functions, such as promoting seed germination (Li et al., 2012), fruit ripening (Muñoz-Vargas et al., 2018), lateral root formation (Mei et al., 2017) and participating in stress response. H_2_S persulfidates proteins to regulate their functions. Chen et al. reported that H_2_S positively regulates abscisic acid signaling through persulfidation of SnRK2.6 in guard cells (Chen et al., 2020). Shen et al. reported that H_2_S persulfidated NADPH oxidase enhancing its ability to produce reactive oxygen species (Shen et al., 2020). Aroca et al. reported that there were 2015 persulfidated proteins, including MPK4, in 30-day-old Arabidopsis WT seedlings (Aroca et al., 2017). In our previous studies, we reported that H_2_S alleviates cold stress through MAPK signals, especially those of MPK4, in Arabidopsis, however, the underlying mechanism remain elusive. In this study, we explored whether MPK4 is the direct target of H_2_S in the alleviation of cold stress and whether this involves the persulfidation of MAPK.

The important roles of MAPKs in H_2_S’s physiological functions have been reported in several studies. The expression level of TaMPK4 increased after NaCl + H_2_S treatment compared with NaCl alone in wheat seedlings (Guo et al., 2019). The MAPK expression level was up-regulated by H_2_S treatment, while it decreased in the presence of an H_2_S biosynthesis inhibitor or H_2_S scavenger in cucumber roots under nitrate-stress conditions (Qi et al., 2019). Here, we used root tip-bending experiments to study the roles of MEK2 and MPK4 in the H_2_S cold-stress alleviation process, and the results showed that the alleviation required MPK4 but not MEK2 (Figure 1). As shown in Supplementary Figure S2 the ability of H_2_S to alleviate cold stress was not impaired in *crlk1*, suggesting that CRLK1 is not required. The results indicated that MPK4 is the target of H_2_S in the process of H_2_S alleviating the damage caused by cold stress.

The physiological range of H_2_S was estimated to be 10–100 μM in health animals and humans (Wang, 2012), therefore we treated the purified-protein of MPK4 and MEK2 with NaHS at 0–100 μM and higher concentration to detect the persulfidation of H_2_S to the MPK4 and MEK2. The results showed that MPK4 was basically persulfidated, and the 100 μM NaHS treatment increased the MPK4 persulfidation level (Figure 2A). MEK2 is an upstream component of MPK4 in cold-signaling transduction, and MEK2 was not persulfidated (Figure 2). Thus, MPK4, not MEK2, is persulfidated by H_2_S, and MPK4 is the direct target of H_2_S-mediated persulfidation. Our results showed that MPK4, GST-MEK2 and GST were basically persulfidated. Endogenous persulfidation can be observed in many proteins without H_2_S treatment, and H_2_S treatment increase the persulfidation level, such as GADPH, Tublin in HEK293 cell (Mustafa et al., 2009), MEK1 in human endothelial cells (Zhao et al., 2014), and RBOHD in Arabidopsis (Shen et al., 2020). We infer that there are multiple Cys residues in the protein, and some of them are persulfidation without H_2_S treatment. H_2_S treatment can persulfidate the Cys at a specific site therefore increase the persulfidation level of the protein.

Zhao et al. reported that H_2_S persulfidated MEK1; therefore, it increased the acitvity of MEK1, leading to PARP-1 activation, which attenuates DNA damage (Zhao et al., 2014). The effects of H_2_S on the activities of other MAPK members have not been reported in plants. Here, our result showed that H_2_S increased the MPK4 kinase acitivity (Figure 3).

We also investigated the Cys sites that were persulfidated in MPK4. Thus, we mutated the Cys residues in MPK4 and performed biotin switch assays using Cys-mutated MPK4. The persulfidation level of MPK4 did not disappeared after the Cys residues were mutated (Figure 4). In most reports, the persulfidation occurred on a single Cys residue. For example, H_2_S persulfidated MEK1 at Cys residue 341 (Zhao et al., 2014), H_2_S persulfidated pyruvate carboxylase at Cys265 (Ju et al., 2015), and mutations of Cys341 and Cys265 abolished the basal MEK1 and pyruvate carboxylase activity levels, respectively. Recently, however, more and more reports have found that persulfidation occurs on multiple Cys residues in a protein. Saha et al. reported that of the 11 Cys in specificity protein 1 (SP1), two were poteintial sites of persulfidation: Cys68 and Cys755 (Saha et al., 2016). Shen et al. reported that CysC44 and Cys205 were persulfiated in L-CYSTEINE DESULFHYDRASE1 (DES1) upon NaHS treatment, and RESPIRATORY BURST OXIDASE HOMOLOG PROTEIN D (RBOHD) is specific persulfidated at both Cys825 and Cys890 (Shen et al., 2020). Thus, the persulfidation modification may occur on more than one Cys in MPK4.

In this study, we investigated the functions of H_2_S in cold stress and explored the underlying mechanisms. We found that H_2_S alleviates cold stress through MPK4, not MEK2 or CRLK1, and that H_2_S increases the MPK4 persulfidation and improve the kinase activity level. However, we did not identify the Cys sites of persulfidation in MPK4, possibly because the persulfidation of MPK4 occurred on two or more Cys residues. In future studies, we will analyze the protein structure of MPK4 and use mass spectrometry to investigate this subject, which will be a very interesting study.

## Data Availability

The raw data supporting the conclusions of this article will be made available by the authors, without undue reservation.
