# Supplementary figures and images for: H2S Persulfidated and Increased Kinase Activity of MPK4 to Response Cold Stress in Arabidopsis
Source: Front Mol Biosci. 2021 Mar 11;8:635470. doi: 10.3389/fmolb.2021.635470 (PMC7991836; doi:10.3389/fmolb.2021.635470)

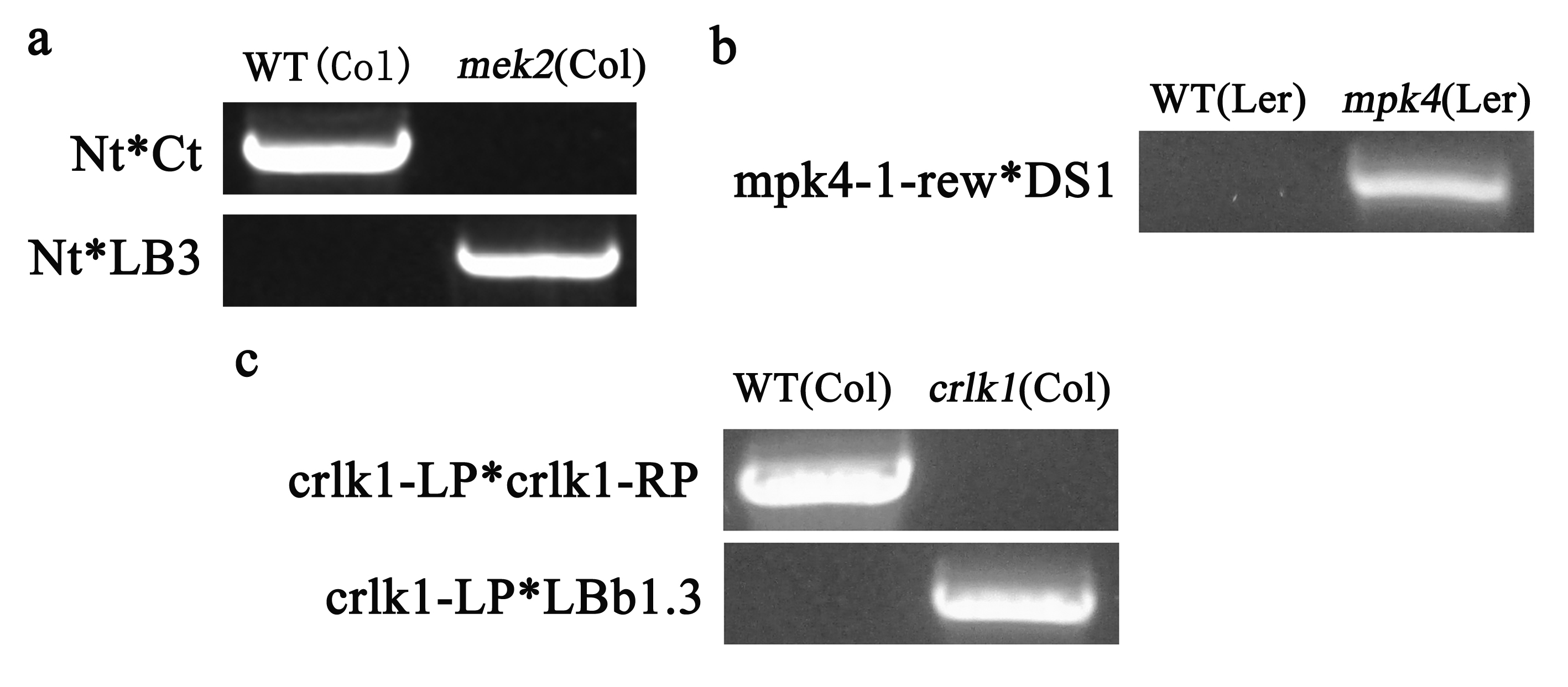

Supplement: Supplementary file 1 [file image1.jpeg]

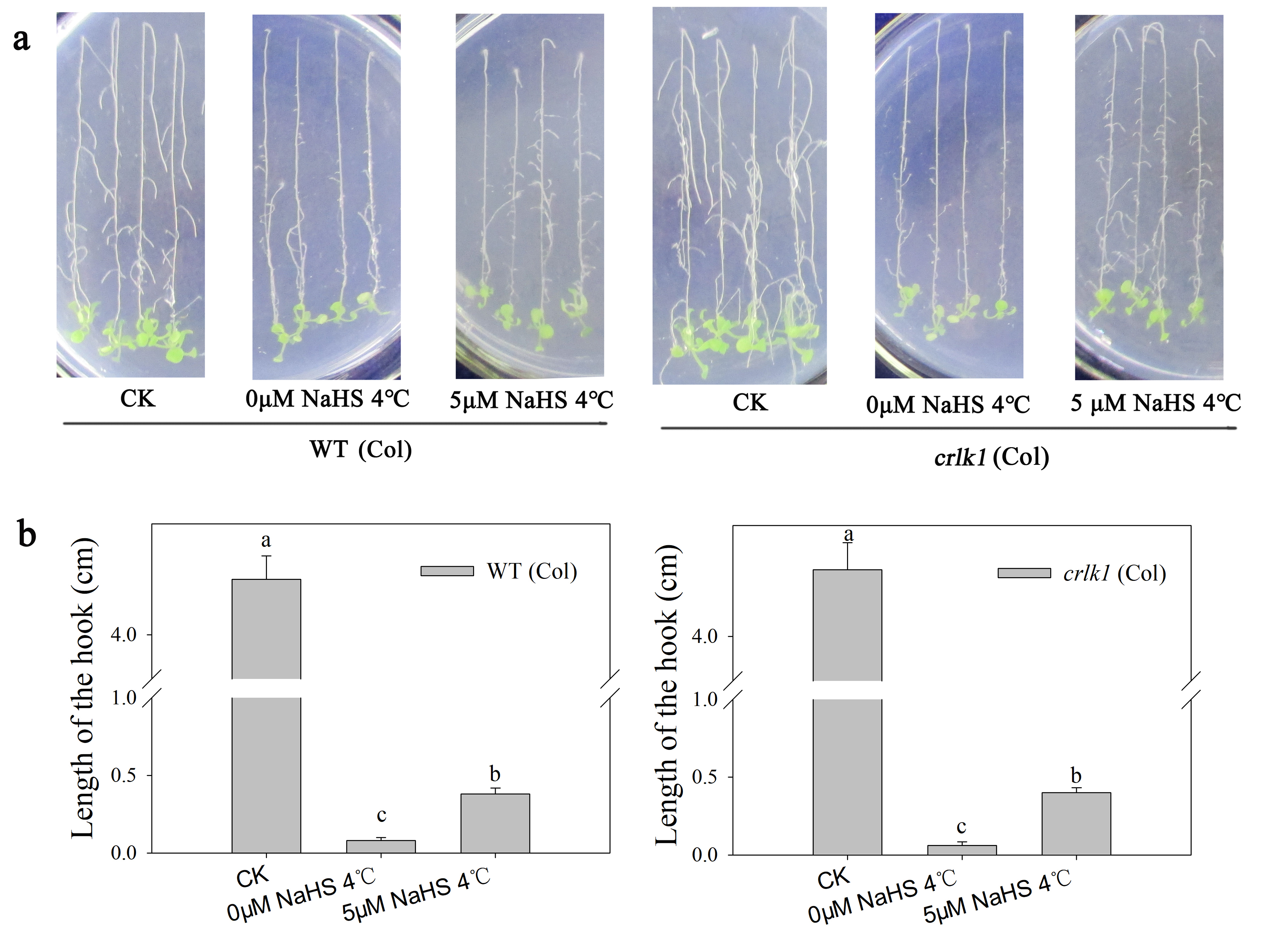

Supplement: Supplementary file 2 [file image2.jpeg]

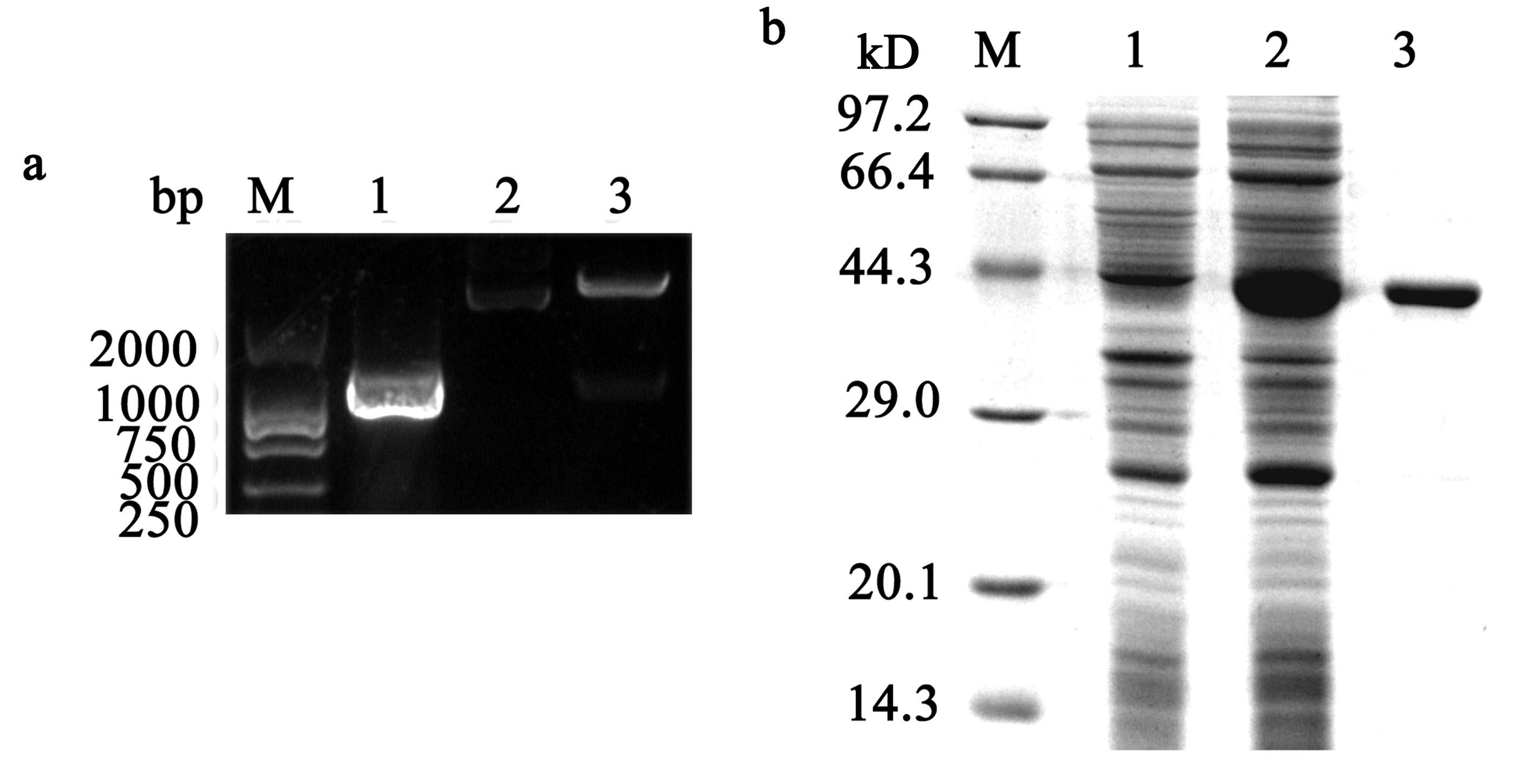

Supplement: Supplementary file 3 [file image3.jpeg]

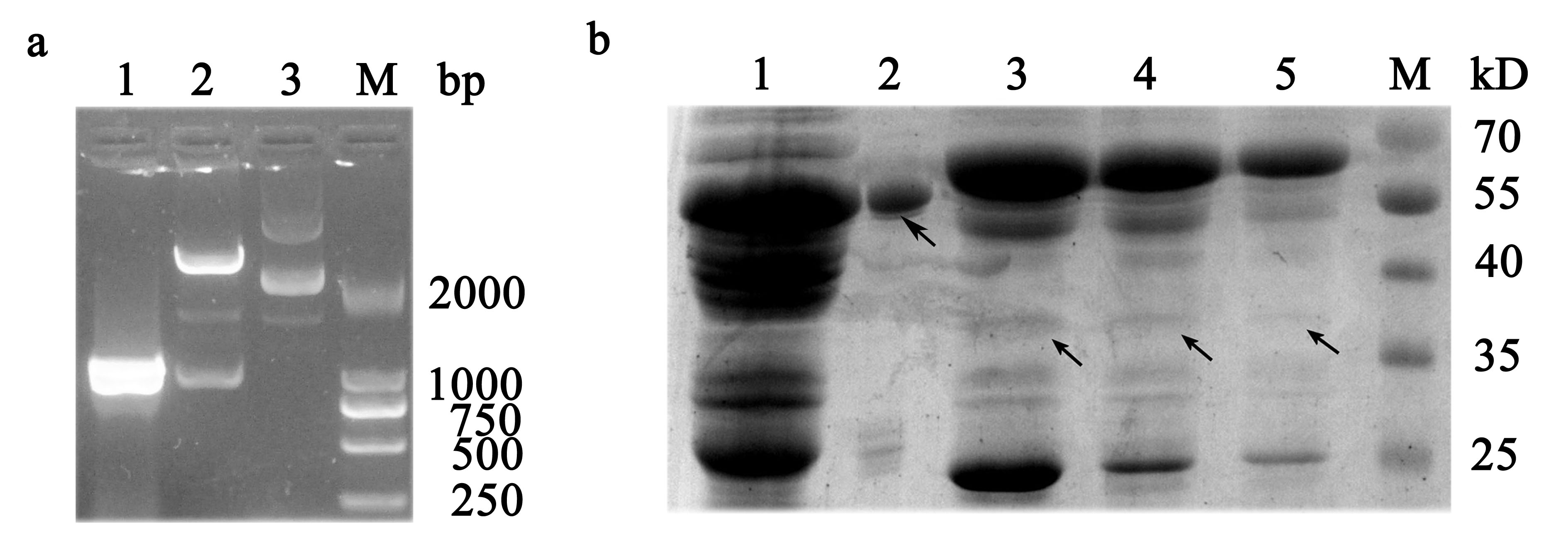

Supplement: Supplementary file 4 [file image4.jpeg]
