# Supplementary material for: H2S Persulfidated and Increased Kinase Activity of MPK4 to Response Cold Stress in Arabidopsis
Source: Front Mol Biosci. 2021 Mar 11;8:635470. doi: 10.3389/fmolb.2021.635470 (PMC7991836; doi:10.3389/fmolb.2021.635470)
Supplement: Supplementary file 5 [file table1.docx]

Table S1 Primers used in this paper

| Primer name | Primer sequence (5'-3') | Purpose | Gene accession number |
| --- | --- | --- | --- |
| mpk4-1-rew | CCAGAGATGCTGATGATTAGT | mpk4 genotyping | AT4G01370 |
| DS1 | GTTTTCGTTTCCGTCCCGCAAG | mpk4 genotyping |  |
| mek2-Nt | ATGAAGAAAGGTGGATTCAGCA | mek2 genotyping | AT4G29810 |
| mek2-Ct | TTACACGGAGAACGTACCAGACA | mek2 genotyping | AT4G29810 |
| LB3 | TAGCATCTGAATTTCATAACCAATCTCGATACAC | mek2 genotyping |  |
| crlk1-LP | TGATGGGGTAAGCTGCTTGCTA | crlk1 genotyping | At5g54590 |
| crlk1-RP | CCCAAAAGAATTGAAAAACAAAATCA | crlk1 genotyping | At5g54590 |
| LBb1.3 | ATTTTGCCGATTTCGGAAC | crlk1 genotyping |  |
| MEK2-F | TGGATCCCCGGAATTCATGAAGAAAGGTGGATTCAGCA | MEK2 CDS amplification | AT4G29810 |
| MEK2-R | GATGCGGCCGCTCGAGTTACACGGAGAACGTACCAGACA | MEK2 CDS amplification | AT4G29810 |
| MEK2EE-M1 | TGCTAAACCTGCCTCGTTTGTC | MEK2 Thr mutant | AT4G29810 |
| MEK2EE-M2 | AGGTTTAGCAAACGAATTTGTGG | MEK2 Thr mutant | AT4G29810 |
| MPK4-F | CAGCCATATGGCTAGCATGTCGGCGGAGAGTTGTTT | MPK4 CDS amplification | AT4G01370 |
| MPK4-R | GCTCGAATTCGGATCCTCACACTGAGTCTTGAGGATTGAAC | MPK4 CDS amplification | AT4G01370 |
| MPK4-M1 | CAGCCATATGGCTAGCATGTCGGCGGAGAGTGCTTTCG | MPK4 Cys mutant | AT4G01370 |
| MPK4-M2 | TTGTAGCAGCAGCGACAATTCCA | MPK4 Cys mutant | AT4G01370 |
| MPK4-M2 | TGGAATTGTCGCTGCTGCTACAA | MPK4 Cys mutant | AT4G01370 |
| MPK4-M3 | GAAAAAACCGAGCATGATCATCAG | MPK4 Cys mutant | AT4G01370 |
| MPK4-M3 | CTGATGATCATGCTCGGTTTTTTC | MPK4 Cys mutant | AT4G01370 |
| MPK4-M4 | AGCTTTAGATCAGCATTTGCATTCA | MPK4 Cys mutant | AT4G01370 |
| MPK4-M4 | TGAATGCAAATGCTGATCTAAAGCT | MPK4 Cys mutant | AT4G01370 |
| MPK4-M5 | TGTGTATTCAGAGGCATTAAGTAGCAG | MPK4 Cys mutant | AT4G01370 |
| MPK4-M5 | CTGCTACTTAATGCCTCTGAATACACA | MPK4 Cys mutant | AT4G01370 |
| MPK4-M6 | TCACCGAGTATAGCACCGACAGA | MPK4 Cys mutant | AT4G01370 |
| MPK4-M6 | TCTGTCGGTGCTATACTCGGTGA | MPK4 Cys mutant | AT4G01370 |
| MPK4-M7 | TATGGGTGGGCCAACGCC | MPK4 Cys mutant | AT4G01370 |
| MPK4-M7 | GGCGTTGGCCCACCCATA | MPK4 Cys mutant | AT4G01370 |
| MPK4-M8 | GGCCTCACAGCTACCGGTTC | MPK4 Cys mutant | AT4G01370 |
| MPK4-M8 | GAACCGGTAGCTGTGAGGCC | MPK4 Cys mutant | AT4G01370 |
